# A Computerized Version of the Scrambled Sentences Test

**DOI:** 10.3389/fpsyg.2017.02310

**Published:** 2018-01-09

**Authors:** Roberto Viviani, Lisa Dommes, Julia E. Bosch, Julia C. Stingl, Petra Beschoner

**Affiliations:** ^1^Institute of Psychology, University of Innsbruck, Innsbruck, Austria; ^2^Psychiatry and Psychotherapy III, University of Ulm, Ulm, Germany; ^3^Research Division, Federal Institute for Drugs and Medical Devices, Bonn, Germany; ^4^Psychosomatic Medicine and Psychotherapy, University of Ulm, Ulm, Germany

**Keywords:** optimism, depression, subsyndromal depression, cognitive processing, neurobiological models of cognition, neurobiological models of optimism, neurobiological models of depression

## Abstract

The scrambled sentences test (SST), an experimental procedure that involves participants writing down their cognitions, has been used to elicit individual differences in depressiveness and vulnerability to depression. We describe here a modification of the SST to adapt it to computerized administration, with a particular view of its use in large samples and functional neuroimaging applications. In a first study with the computerized version, we reproduce the preponderance of positive cognitions in the healthy and the inverse association of these cognitions with individual measures of depressiveness. We also report a tendency of self-referential cognitions to elicit higher positive cognition rates. In a second study, we describe the patterns of neural activations elicited by emotional and neutral sentences in a functional neuroimaging study, showing that it replicates and extends previous findings obtained with the original version of the SST. During the formation of emotional cognitions, ventral areas such as the ventral anterior cingulus and the supramarginal gyrus were relatively activated. This activation pattern speaks for the recruitment of mechanisms coordinating motivational and associative processes in the formation of value-based decisions.

## Introduction

The Scrambled Sentences Test (SST) was introduced in the early' 90s to unmask latent depressogenic schemas (Wenzlaff, 1991). Participants were asked to form a sentence from a set of words and write it down, thus mimicking the process of generation of cognitions. Negative cognitions could be elicited by allowing them among the alternative sentences that could be formed from the set.

The rationale of the SST was grounded in the hypothesis that depressogenic schemas were causally active in precipitating episodes of depression (Beck, 1963, 1987). Contrary to initial expectations, this hypothesis was proving difficult to verify empirically. Healthy controls and remitted depressives, who are known to be at higher risk to relapse (Kendler et al., 2000), displayed the same amount of negative cognitions (Lewinsohn et al., 1981; Hamilton and Abramson, 1983; Gotlib and Cane, 1987; for a discussion, see Clark et al., 1999). Wenzlaff and Bates (1998) suggested that this may have been explained by remitted depressives actively suppressing negative cognitions by recourse to effortful, control processes of executive nature. They therefore proposed to investigate cognitions with the SST under the administration of a simultaneous cognitive load to unmask the latent tendency. Over the years, several studies have been carried out with the SST. They have consistently shown its sensitivity to assess vulnerability to depression after matching for self-reported depressive symptoms (Hedlund and Rude, 1995; Wenzlaff and Bates, 1998; Rude et al., 2002, 2003, 2010).

Apart from the results on risk groups, the SST consistently showed that the healthy tend to avoid negative cognitions. Importantly, this result may also be obtained without cognitive load (Hedlund and Rude, 1995; Viviani et al., 2010). Furthermore, the amount of negative cognitions formed without cognitive load correlates with scores in standard scales of depressiveness (Viviani et al., 2010). Therefore, while the tendency to suppress negative cognitions by activating cognitive control processes of executive nature might play a role in depressive individuals or in individuals with a latent predisposition to depression, a different mechanism seems to be active in the healthy to regulate the occurrence of negative cognitions. The independence of this mechanism from executive processes is likely since it is not modulated by individual differences in working memory capacity, nor appears to recruit cortical areas associated with executive function in neuroimaging studies (Viviani et al., 2010). This suggests that without cognitive load the SST may sensitive to spontaneous mechanisms of emotion regulation, i.e., mechanisms that do not rely on executive function to regulate processing of emotional content (Gyurak et al., 2011; Viviani, 2013; Messina et al., 2016).

Here, we describe the development of a computerized version of the SST, focusing on the spontaneous condition (i.e., in the absence of a cognitive load). Our aim is the development of a test that exploits the advantages of computerized testing for administration in large samples and its use in functional magnetic resonance imaging (fMRI) studies. The absence of a simultaneous cognitive load is a convenient condition to study the neural substrates of the mechanisms through which negative cognitions are avoided, since the cognitive load would generate its own confounding activations in an fMRI study. In this paper, we first characterize the changes made to the original SST to adapt it to a computerized version that simultaneously collects data on the valence of the cognitions formed during the test. Second, we provide data on the relative frequency of negative cognition scores obtained with our computerized version in a sample of healthy young adults. The minimal requirement for a computerized version of the SST to be validated is the reproduction of the tendency to avoid negative cognitions. Another important requirement is the association with depressiveness from rating scales. Even when not showing clinical depression, samples from the general population show variance in reports of depression symptoms, a condition sometimes referred to as subsyndromal or subthreshold depression (Judd et al., 1997). Subsyndromal depression is a condition of considerable interest, being associated with significant impairments and risk for full-fledged depressive episodes (Judd et al., 1996; Lewinsohn et al., 2000). Here, this variability was used to test the sensitivity of the SST to depressiveness without prejudice to the issue of cognitive control in clinical depression. Third, we report on a functional MRI study in which we replicate the findings of Viviani et al. (2010), which used the SST in the original form. In that study, the only modification of the original SST was that participants could not write the sentence in the scanner (which would lead to artifacts). Participants would move on from one sentence to the next, so that neither the outcome nor the precise timing of trials could be recorded. The computerized version of the SST aims at overcoming these difficulties. That study indicated that specific areas, notably the ventral anterior cingulated/ventromedial prefrontal cortex (vACC/VMPFC) and the supramarginal gyrus (SMG) were associated with the formation of emotional sentences. In the discussion, we will comment on our findings and the possible relevance of the SST to investigate the relation between motivational states and cognition.

## Materials and methods

### The scrambled sentences test

The SST consists of a series of trials, in which a set of words are presented to participants: the scrambled sentence. The participants must mentally compose a syntactically correct sentence by reordering the words. For each scrambled sentence, two possible sentences can be formed. For example, the scrambled sentence “June in falls birthday her July” may be recomposed into the sentence “her birthday falls in June” or the sentence “her birthday falls in July”. The choice of the sentence implicitly excludes one word from the scrambled set (June or July). Below, we refer to these two words determining the chosen sentence as the “targets.” The scrambled sentences are designed so that the targets occupy the last position in a syntactically correct sentence. Participants indicate their choice on the basis of the position on the screen of the last word in the sentence they formed, which is a target. If the participant chose “her birthday falls in June,” and the word “June” is on the left of the midline, then she presses a left button. The right button signals choice of the other sentence. A vertical bar in the lower half of the screen facilitates locating the midline. To elicit the tendency to prefer positive sentences, the scrambled sentence is designed to force an implicit choice between negatively/pessimistic and positively/optimistic toned sentences. One example is “the future is bright/dismal.” Note that we use the term “scrambled sentence” to refer to the set of words, and “sentence” to a possible choice of the participant.

### Computerized version of the scrambled sentences test

**Figure 1:**
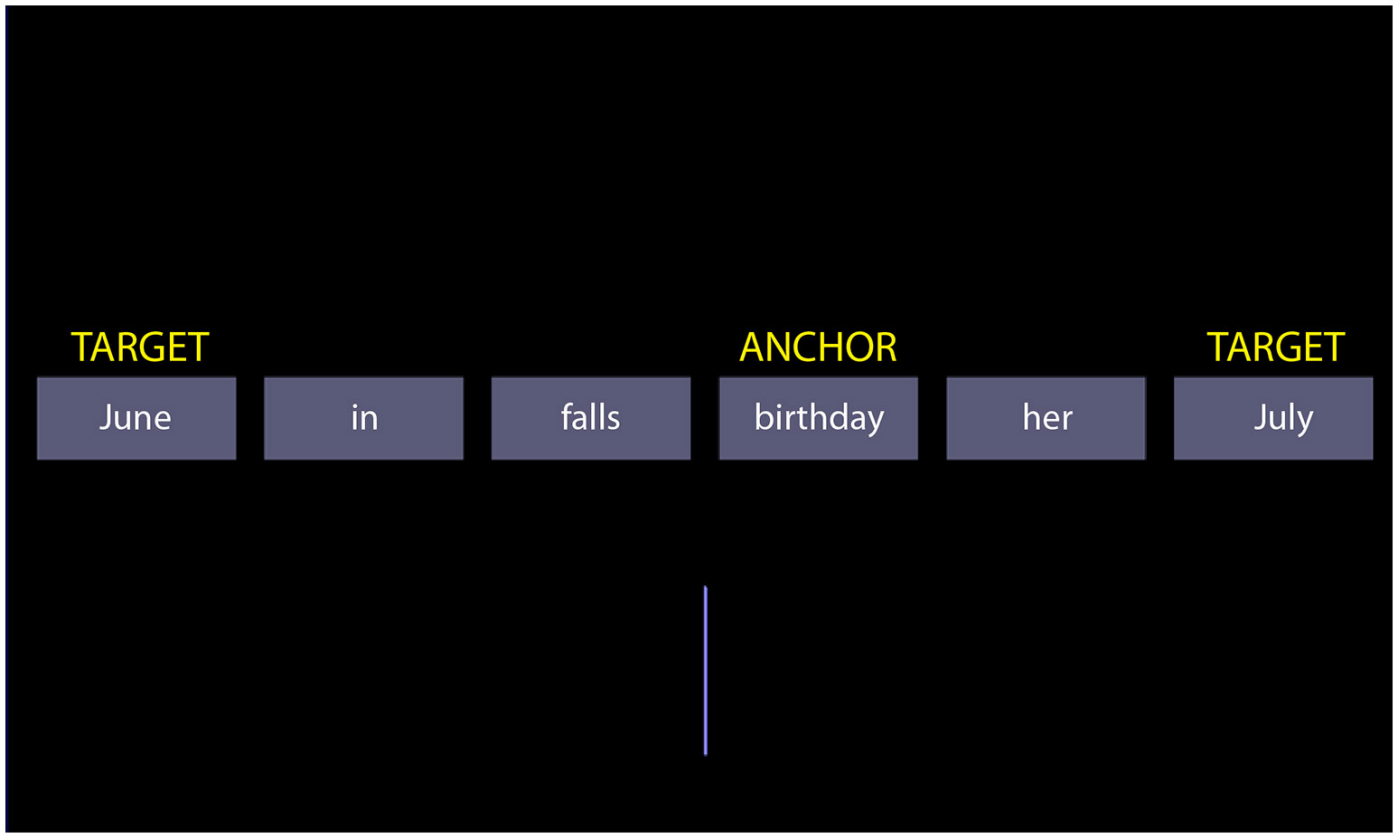
A screenshot of the scrambled sentences task. The yellow labels are not shown during the test; they exemplify the terminology used in the text to refer to particular words of the scrambled sentence.

In the computerized version of the SST, the words were presented on-screen (Figure 1). Furthermore, the scrambled sentences were standardized in several respects. First, all scrambled sentences were composed of six words. This made the sentences more akin to each other, and avoided the screen being redesigned at the beginning of trials in which the number of words in the word changed. The redesign may introduce arousal confounding effects, or visual novelty effects in an fMRI study. (The exemplary scrambled sentences mentioned here may appear not to follow these rules because we report English translations of the German sentences we used in the study).

Second, as already noted, in our computerized version of the SST the targets were always the last possible word in the sentence (as in: the future is dismal/bright). This constraint allows collecting the response of the user using an instruction that only refers to the location of the last word in the sentence. This makes the criterion for indicating the choice independent from the criterion distinguishing between sentence alternatives (that is, when instructing participants no reference is made to content that may influence the choice of the sentence). Furthermore, unless it is clear from the outset that participants may report their choices by indicating the location of the last word in the sentences, they have the additional task to determine where the target words are located in the array of presented words. This is an extraneous task that would introduce an additional working memory load. It should be noted, however, that according to the rules of the German language it is possible to construct sentences in alternative ways, although some constructions sound somewhat odd. For example, unlike in the English language, there is no constraint for the subject to come first in the sentence. In such cases, it is legally possible to put the target at the beginning, albeit with somewhat awkward results. Therefore, the detection of the chosen sentence should be viewed as contaminated by some degree of noise (the degree of this contamination may be language specific). The scrambled sentences of the original SST test (Wenzlaff, 1991) that could not be adapted to these two criteria for the computerized version were discarded and replaced by new sentences.

We were also concerned with characterizing the scrambled sentences thematically. In this study, the scrambled sentences belonged in two factors, reflecting different possible influences on sentence choice. One factor referred to the semantic content of the sentences. One group of scrambled sentences in this factor reflected statements about success/failure, or more generally the dichotomy of optimism/pessimism. This theme refers to cognitions about the future (the sentence about the bright/dismal future is a prototypical example of this group). Another group of scrambled sentences, while still usually involving statements about the future, specifically involved themes of attachment/rejection and loss. Loss and rejection is a common theme in depression and characterizes life events likely to trigger episodes (Keller et al., 2007). An example of a scrambled sentence in this group was “there is plenty of hope/mourning in life.” A third and final group contained neutral sentences of no emotional relevance (such as the sentence about the birthday). The second factor was the self-referential nature of the scrambled sentence. In non-self-referential sentences, the statement concerned a third person (“her birthday falls in June/July”). In the self-referential variant, the sentence concerned the self (“my birthday falls in June/July”). These two versions were obtained by systematically varying subject or possessive pronouns, keeping the rest of the words of the scrambled sentence unchanged. The intended use of this factor was to assess cognitions about the self, which are, together with cognitions about the world and the future, important aspects of cognitions affected by mood (Beck, 1976). Self-referential negative cognitions, such as self-blame, are also common in severe depression (Pulcu et al., 2013). Furthermore, self-relevant content may be more effective in eliciting a measure of predisposition to depression (Johnson et al., 2007).

Each of the six cells of the factorial design contained 12 scrambled sentences. In addition, there were eight additional scrambled sentences where the targets were both located on the same side of the screen. In these trials, the choice of the user is not informative of the chosen sentence, but allows verifying that the user is carrying out the task. We refer to these trials below as “sentinel trials.” In total, there were 72 scrambled sentences in the regular trials and eight scrambled sentences in the sentinel trials. Participants had 12 s to give their response.

Targets were chosen to match for length and frequency in the written corpus of the German language (Institut für Deutsche Sprache., 2013). Target word frequency was matched between the levels of the two factors [*F*_(5, 66)_ = 0.194, *p* = 0.9638—this implies matching between emotional and neutral trials and between self-referential and non-self-referential trials] and in positive, negative, and neutral target valence [*F*_(2, 70)_ = 0.754, *p* = 0.474]. Similarly, word length of targets was matched between levels of the factors [*F*_(5, 66)_ = 0.1458, *p* = 0.9806] and target valence [*F*_(2, 70)_ = 0.1335, *p* = 0.8753]. The position of the targets was chosen to be identical between the levels of the factors, and in non-neutral trials the positive and negative alternatives appear exactly equally often on the left and on the right, with respect of their position in the scrambled word set from left to right, with respect of their distance to the midline. This ensured that there could be no confound between position and choice of sentence based on the semantic quality of the targets. The position of targets on the right or left was constant for a given scrambled sentence across the experiment.

Besides the targets, another important word in the scrambled sentence is the “anchor.” The anchor is the word to which targets refer (often, but not invariably, the subject of the sentence; see section Introduction). In the example of Figure 1 “birthday” is the anchor. In sentences where the semantic or predicative link of the targets is missing, the anchor is the grammatical subject of the sentence. In addition to the match concerning length and frequency of targets, exact match across factor levels was obtained with respect to the position of the anchor, its distance to the midline, and the distance of targets to the anchor. This latter match ensured that there was no confound between the distance to the anchor and semantic quality of the target in the determination of target choice (it is conceivable that targets closer to the anchor be chosen more often). Although perhaps of less importance, matching of anchors was also checked. The anchors were matched for frequency [*F*_(5, 66)_ = 0.1183, *p* = 0.9879] and for word length [*F*_(5, 66)_ = 0.6496, *p* = 0.6628] across factor levels.

Another dimension of matching concerned all words (not only anchors and targets) in the word set of each trial. Here too, both frequency [*F*_(5, 66)_ = 0.2102, *p* = 0.957] and size [*F*_(5, 66)_ = 0.3223, *p* = 0.8979] were matched between the levels of the design. Sentences were also matched between levels for aggregate frequency [*F*_(5, 66)_ = 0.359, *p* = 0.8743] and aggregate size [*F*_(5, 66)_ = 0.4101, *p* = 0.8401]. These match ensured that the overall frequency of words used in a scrambled sentence as well as their total length do not differ between the levels of the design, thus avoiding a confound ensuing in the process of composing the sentence as a whole. The scrambled sentences are reproduced in the Appendix.

## Behavioral study

### Participants

Participants (*N* = 53) were recruited from students at the Institute of Psychology of the University of Innsbruck as part of their requirements to complete their study. Participants were asked to complete the CES-D questionnaire, a self-rating instrument used in epidemiological studies of depressiveness (Radloff, 1977, German version: Hautzinger and Bailer, 1993). Participants also completed a standard questionnaire for anxiety (STAI, Spielberger et al., 1967, German version: Laux et al., 1981) and for anhedonia (SHAPS-D, Snaith et al., 1995, German version: Franz et al., 1998).

### Methods and statistical analysis

The task was coded in Visual Basic (Microsoft, Redmond, WA) running on a laptop computer.

Data were analyzed in R (R Core Team, 2015) with the lme4 package (function glmer, Bates, 2010) to model positive sentences in a mixed-effects logistic regression with scrambled sentences and subjects as random effects. Significance levels given below are two-tailed, unless otherwise specified. One-tailed significance values were chosen when there was an a priori hypothesis on the directionality of the test.

### Results

Data were collected from 53 participants, all psychology students at the University of Innsbruck. We first checked the number of errors made in the sentinel trials. There were 31 participants with no errors in the sentinel trials, 12 made one error, and 5, 4, and 1 participants made 2, 3, and 4 errors, respectively. In the analyses that follow, we excluded participants who made more than one error in the sentinel trials (additional analyses that included only participants that had made no errors did not differ qualitatively from those reported here. Errors were made most often in two sentinel sentences that appeared to be unscrambled in unconventional ways in a minority of participants). The final selected sample included 43 participants (29 women, mean age 23.7, std. dev. 3.0). Being university students, all participants had a high-school diploma. Mean depressiveness was 14.5 (CES-D; std. dev. 7.76, range 3 to 35). Mean anxiety scores were 38.1 for state (STAI-S, std. dev. 8.99, range 24 to 67) and 39.6 for trait (STAI-T, std. dev. 8.8, range 24 to 60); anhedonia scores averaged 33.3 (SHAPS-D, std. dev. 5.1, range 20 to 42).

The first question of interest was whether participants formed more positive than negative sentences. There were on average 25.4% negative sentences, a result that is concordant with expectations from the pen-and-paper form of the test. This ratio strongly contradicted a null hypothesis of 50% sentences of either type (logistic regression, *z* = 7.79, *p* < 0.001). Both types of sentences (optimism/pessimism and rejection/loss) contributed equally often to this tendency (*z* = 0.44, *p* = 0.66). In contrast, there was an association of positive sentence rates with the self-referential character of the scrambled sentences. A scrambled sentence like “my future is bright/dismal” elicited more positive sentences than “the future is bright/dismal” (*z* = 2.19, *p* = 0.014, one-tailed, corresponding to a fitted increase of 9% in the formation rate of positive sentences in the self-referential set).

**Figure 2:**
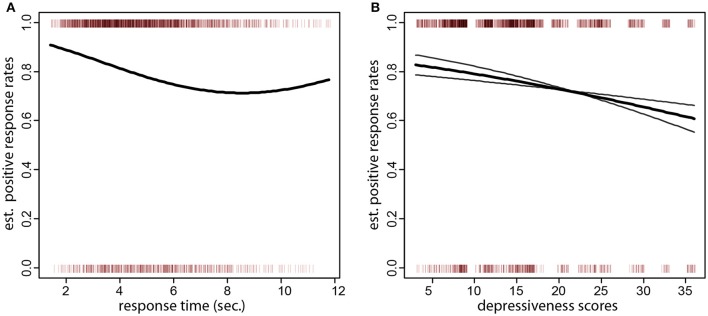
**(A)** Sentence type (negative or positive) over response times (x axis). The small vertical bars at the top and bottom of the plot denote the individual sentences (positive at the top of the plot and negative at the bottom). The thick black line is the fitted formation rate of positive sentences as a function of response times. One can also see that the bulk of all responses were given within a response time of 7 s. **(B)** Sentence type (negative or positive) over depressiveness scores (x axis). The thick black curve is the fitted formation rate of positive sentences as a function of depressiveness scores (x axis). The gray curves are the fitted probabilites for pessimistic/optimistic and loss thematic groups of the scrambled sentences (the probability for the pessimistic/optimistic starts higher and ends lower). One can see that individuals with low depressiveness scores have over 80% fitted positive sentence rates. The rates drop to about 70% in individual with scores larger than 30. This effect is more pronounced in the pessimistic/optimistic scrambled sentences set, where the rates drop to about 60%.

A desirable property of the test was that variables related to the composition of the trial did not influence the formation of positive sentences. Specifically, there was no such association with the length or frequency of the chosen target word (*z* = 0.92, *p* = 0.36; *z* = −1.27, *p* = 0.26), the position of the chosen target word in the array (*z* = 0.86, *p* = 0.39), or the cumulative length or frequency of the words in the scrambled set (*z* = 1.22, *p* = 0.22; *z* = −0.72, *p* = 0.47). Similarly, no association was found between the rate of positive sentences and the length, frequency, or position in the word array of the anchor (*z* = −1.29, *p* = 0.19, *z* = 1.40, *p* = 0.16, *z* = −0.48, *p* = 0.62). However, there was a negative association of positive sentences with the time participants took to respond (i.e., longer thinking times produced more negative sentences, *z* = −3.19, *p* = 0.001, quadratic term, *z* = 2.29, *p* = 0.02, corresponding to a fitted 2.8% lower formation rate of positive sentences per second). As one can see in Figure 2A, the rate of positive sentences decreased up to about 7 s. response times, after which it leveled off.

The second question of interest was the negative association of the rate of positive sentences ratio with depressiveness scores. From the pen-and-paper version, we expected individuals with higher depressiveness scores to form more negative sentences. This expectation was confirmed by the data (logistic regression of positive sentences on CES-D scores, *z* = −2.31, *p* = 0.010, one-tailed; Figure 2B, thick black curve). The fitted difference in the formation of positive sentences between the lowest and highest quartile of depressiveness scores (8 and 19 CES-D scores) was 6.0%. The association persisted also when depressiveness scores over the highest quartile of the sample were removed from the dataset (i.e., including only participants with CES-D scores 19 or less; *z* = −1.60, *p* = 0.050, one-tailed). Even if both types of scrambled sentences gave rise to positive choices, there was an interaction of sentence type and depressiveness (*z* = −2.37, *p* = 0.018). Scrambled sentences referring to separation and loss were much less sensitive to depressiveness scores than sentences on optimistic outcomes. When examined separately (Figure 2B, gray curves), the effect of depressiveness scores was not significant in scrambled sentences on separation and loss (*z* = −1.07, *p* = 0.14, one-tailed) but highly significant in the optimism set (*z* = −2.96, *p* = 0.002, one-tailed).

Among the other scales used in the study, there was a trend negative association of positive sentences with anxiety, as measured with the STAI questionnaire (*z* = −1.64, *p* = 0.05, one-tailed). However, this association disappeared when depressiveness scores were included in the model (*z* = −0.36, n.s.). The association with anhedonia scores (measured with the SHAPS-D questionnaire) was not significant (*z* = 0.735, n.s.).

Finally, we examined the response times. Mean response was 4.89 s. (median 4.75, interquartile range: 3.89–5.7 s.) There were only two significant predictors of response times: the self-referential character of the scrambled sentence [associated with shorter responses, *t*_(71)_ = −2.01, *p* = 0.04] and the length of the scrambled sentence [associated with longer responses, *t*_(71)_ = 2.23, *p* = 0.02]. Of particular interest, there was no significant association of response times with depressiveness scores in the emotional set (*t* = −0.44, n.s.) and between set types (emotional or neutral, *t* = 0.87, n.s.).

## Neuroimaging experiment

### Participants

Participants were recruited through announcements publicly displayed at posting board of the local university, and gave their written consent to the neuroimaging study after being informed about the finalities of the study and modalities of the procedure. Exclusion criteria were medical, neurological, or psychiatry pathology (assessed through an interview by a clinical psychologist) and the conditions commonly preventing access to the scanner (such as metal implants, pacemakers, extensive tattoos, pregnancy). The study comprised 33 participants; of these, six committed more than one error in the sentinel trials (three participants committed two errors, two participants three errors, and one participant four errors) and were excluded from the analysis. The remaining *N* = 27 participants were of mean age 23.2 years (std. dev., 2.7, 11 females); 20 had received high-school education.

### Methods

The task was adapted for the neuroimaging study by imposing a time limit of 7 s. in responses (determined on the basis of the results of the behavioral experiment, see below). This limit is a technical requirement arising from the necessity to obtain the same task duration in all subjects to plan the MRI scan appropriately. In other respects, the task was identical to the version used in the behavioral study.

Participants were scanned on the premises of the Psychiatry and Psychotherapy Clinic III of the University of Ulm. Prior to scanning, participants were familiarized with the task by completing a short run with unrelated scrambled sentences. MRI data were acquired in a 3-Tesla Magnetom Allegra scanner (Siemens, Erlangen, Germany) equipped with a head volume coil with padding to minimize head motion. Participants viewed stimuli through goggles masking the whole field of vision (Resonance Technology Inc., Northridge, CA) displayed through a script written with the software package Presentation (version 11.0, Neurobehavioural Systems Inc. Albany, CA). Images were obtained using an echo-planar imaging sequence in transversal orientation (TR/TE 2460/35 ms, flip angle 90°, voxel size 3 × 3 mm, slice size 3 mm with a gap of 0.75 mm between sized). For each image, 38 slices were acquired parallel to the AC-PC plane for whole brain coverage. After discarding the first six volumes to allow for equilibration effects, 358 volumes were acquired for a scanning duration of about 14 min 40 s.

### Statistical analysis

Data were realigned, stereotactically normalized into the Montreal Neuroimaging Institute (MNI) standardized space, and resampled to an isotropic voxel size of 2 mm using the SPM8 package (Wellcome Department of Cognitive Neurology, London: online at http://www.fil.ion.ucl.ac.uk). After normalization, a smoothing kernel of 8 mm was applied. The SPM8 package was also used to model trials (emotional and neutral) at the first level (i.e., in the separate datasets from each participant) by creating a regressor convolving an indicator function for the trial with a standard blood oxygen-level dependent (BOLD) response curve. At the first level, realignment parameters were included as nuisance regressors. The fit was obtained by modeling residuals with an AR(1) autocorrelation model, and computed for each voxel separately (Friston et al., 1995). Contrasts of interest of the estimated coefficients of the fit from the first level were taken to the second level for group analysis with subjects as a random effect to account for repeated measurements. At the second level, significance level with peak-level and cluster-level correction (cluster defining threshold *p* < 0.005) were computed with a permutation test (6,000 resamples; Holmes et al., 1996). Overlays were produced with the freely available software MRIcron (http://people.cas.sc.edu/rorden/mricron/index.html). Designations of cortical areas in tables were obtained from the “aal” atlas (Tzourio-Mazoyer et al., 2002) provided with this software package.

The study was approved by the Ethical Committee of the University of Ulm (approval date 10.11.2014, application nr. 290/14).

### Results

The behavioral data in the neuroimaging experiment (*N* = 27) replicated the results of the previous larger validation experiment. Here, participants had 7 s. to give their response and were informed of this fact. Participants made more positive sentences in the emotional set (19% negative sentences, mixed effects logistic regression, *z* = 7.67, *p* < 0001). Self-referential versions of the scrambled sentences were more effective in eliciting positive sentences (*z* = 2.03, *p* = 0.02, one-tailed), while content type did not differ in this respect (*z* = 0.48, n.s.). Reaction times were also negatively associated with positive responses (*z* = −3.15, *p* = 0.001, one-tailed), as in the previous dataset. In comparison to the behavioral experiment, there was no quadratic term in the reaction times reflecting the tail-off of the decrease in positive responses as here responses had to be given within the time limit. Reaction times did not differ in the emotional and neutral sets (mixed effects linear regression, *t* = −0.32, n.s.) and averaged 4.01 s. (this shorter mean reaction time, due to the response time limit, accounts for the lower proportion of negative sentences relative to the previous experiment. The fitted proportion of negative sentences in the previous experiment for trials with reaction time 4 s. is about 20%: see Figure 2A). Misses (failure to indicate a sentence after the 7 s. time limit) occurred infrequently (in about 1.4% of trials on average) and were slightly more frequent in the emotional set (0.7% more frequent), but this difference was not significant (mixed effects logistic regression, *z* = 1.17, *p* = 0.24). Overall, these data indicate that participants were responding to the test similarly in the behavioral setting of the previous experiment and in the scanner.

**Figure 3:**
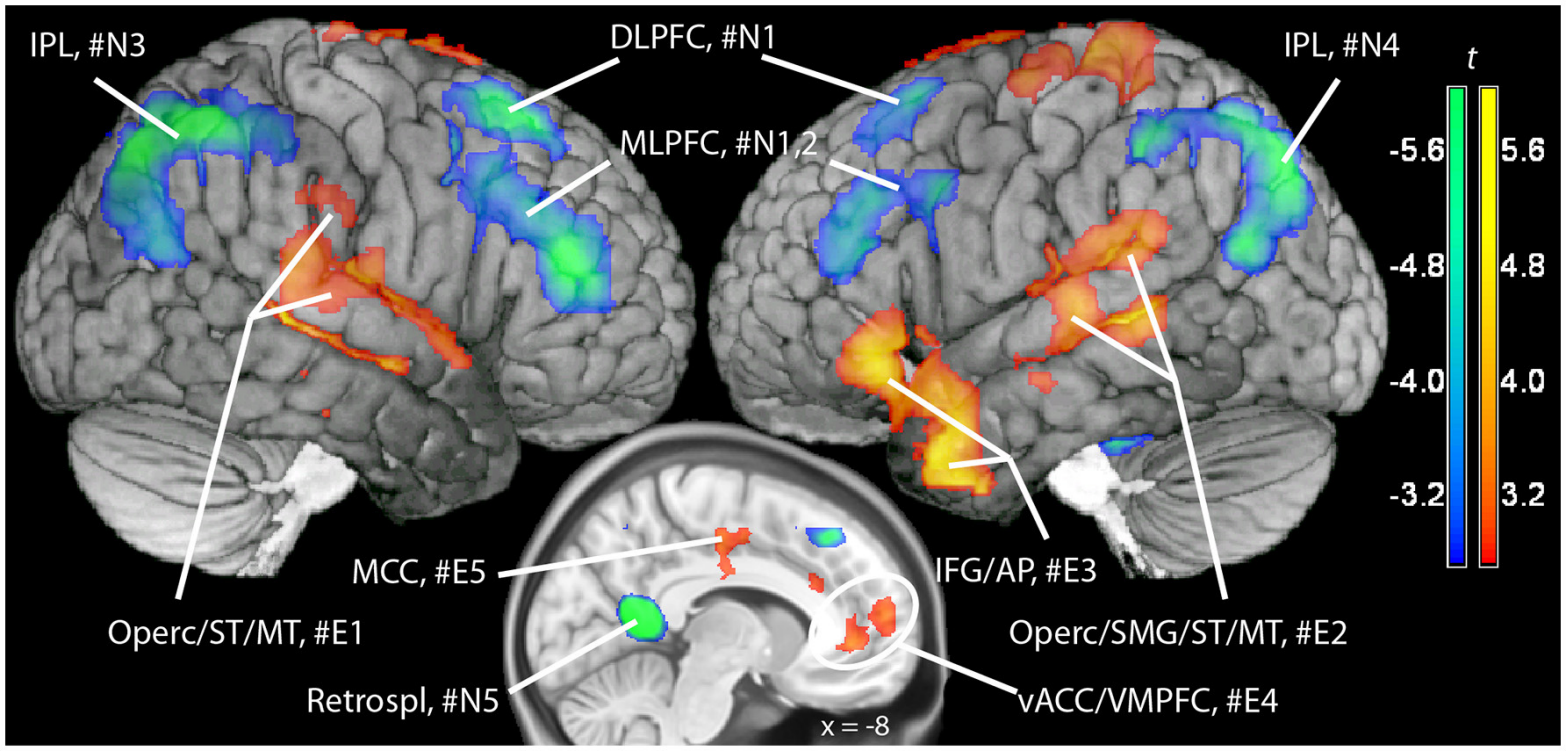
Maps of t statistics obtained in the emotional vs. neutral contrast (in red-yellow) and in the neutral vs. emotional (in blue-green) rendered on a template brain. Data thresholded for ilustration purposes at *p* < 0.005, uncorrected. The codes introduced by # refer to the cluster designations in Tables 1, 2. IPL, inferior parietal lobe; DLPFC, dorsolateral prefrontal cortex; MLPFC, mediolateral prefrontal cortex; Operc, parietal operculum; SMG, supramarginal gyrus; ST, MT, superior, middle temporal gyrus; MCC, middle cingular cortex; Retrospl, retrosplenial cortex/posterior cingular cortex; IFG, inferior frontal gyrus; AP, temporal pole; vACC/VMPFC: ventral anterior cingulus/ventromedial prefrontal cortex.

In a previous neuroimaging study in which participants formed the sentences mentally, a shift of activity from dorsal to ventral prefrontal areas was observed between the neutral and the emotional set (Viviani et al., 2010). Our objective was to verify that a similar shift of activity occurred with the computerized version of the test. Relative to the emotional set, the neutral set activated dorsal prefrontal areas associated with cognitive control and executive function (dorsolateral prefrontal cortex, DLPFC, dorsal medial prefrontal cortex, DMPFC; Figure 3, in blue-green, and Table 1). Significant activity in this comparison was also noted in the inferior parietal lobe, the parahippocampal cortex, and the retrosplenial cortex. Relative to the neutral set, the emotional set activated the vACC/VMPFC, the SMG/rolandic operculum and the middle temporal gyurs, and the inferior frontal gyrus (Figure 3, in red-yellow, and Table 2). Significant activity in this contrast was also noted in the left anterior temporal pole, the middle cingular gyrus, and in an area spanning the precentral and postcentral gyri.

**Table 1:** Neutral vs. emotional set.

**Cluster #**	**Brain area**	**MNI Coord**.	***t***	***p* (peak)**	***k***	***p* (cl.)**
N1	R Frontal Sup (DLPFC, BA8)	24 28 50	−7.32	<0.001	4658	0.002
	R Frontal Sup (DLPFC, BA8)	22 20 54	−7.27	0.001		
	R Frontal Mid (MLPFC, BA8,9)	30 16 52	−5.91	0.031		
	R Frontal Mid/Inf (MLPFC, BA45)	48 38 18	−6.55	0.007		
	Suppl motor (DMPFC/dACC, BA8)	6 24 50	−7.11	0.002		
	L Frontal Sup (BA8)	−20 14 56	−5.33	0.105		
	L Frontal Mid (BA8,9)	−28 18 42	−5.06	0.171		
N2	L Precentral (BA44)	−42 6 32	−6.65	0.006	1335	0.025
	L Frontal Mid (MLPFC, BA45)	−40 30 22	−5.02	0.180		
N3	R Angular (IPL, BA40)	44 −56 54	−7.89	<0.001	2923	0.007
	R Angular (IPL, BA7)	36 −74 46	−6.49	0.009		
	R Supramarg (IPL, BA40)	44 −36 36	−4.65	0.33		
N4	L Occipital Mid (BA19)	−34 −82 40	−6.26	0.016	2452	0.008
	L Parietal Inf (IPL, BA7)	−28 −70 40	−6.20	0.018		
	L Occipital Sup (BA7,19)	−34 −78 48	−5.58	0.063		
N5	L Precuneus/Retrospl. (BA30)	−6 −56 10	−11.02	<0.001	1432	0.022
	R Precuneus/Retrospl. (BA30)	8 −56 10	−6.90	0.003		
N6	L Frontal Inf Orb (BA47)	−32 38 −14	−6.57	0.006	371	0.146
N7	R Frontal Inf Orb (BA47)	32 38 −12	−8.53	<0.001	219	0.264
N8	L Fusiform (BA37)	−30 −36 −22	−9.80	<0.001	935	0.041
	L Parahippocampal (BA36)	−22 −14 −26	−4.20	0.398		
	L Parahippocampal (BA20)	−32 −24 −24	−3.90	0.266		
N9	R Parahippocampal (BA30)	26 −30 −26	−7.28	0.001	751	0.057
	R Parahippocampal (BA30)	20 −30 −16	−5.70	0.047		
N10	L Temporal Inf (BA37)	−54 −56 −14	−8.65	<0.001	355	0.153

*Explanation of symbols. MNI Coord, Montreal Neurological Institute coordinates; in mm; t, Student's t; p (peak), significance level; peak-level correction; k, cluster size; in 2 × 2 × 2 voxels; p (cl.): significance level, cluster-level correction. R, L, right, left; Sup, Mid, Inf, superior, middle, inferior; Suppl, supplementary; Supramarg, supramarginal; Retrospl, retrosplenial. DLPFC, dorsolateral prefrontal cortex; MLPFC, mediolateral prefrontal cortex; DMPFC, dorsomedial prefrontal cortex; dACC, dorsal anterior cingulate; IPL, inferior parietal lobe*.

**Table 2:** Emotional vs. neutral set.

**Cluster #**	**Brain area**	**MNI Coord**.	***t***	***p* (peak)**	***k***	***p* (cl.)**
E1	R Temporal Mid (BA37)	42 −42 0	6.12	0.020	2560	0.008
	R Temporal Mid (BA21)	48 −36 2	6.04	0.024		
	R Temporal mid (BA20)	50 −32 −12	5.34	0.093		
	R Rolandic Operc (BA48)	50 −14 14	4.84	0.238		
E2	L Temporal Mid (BA21)	−50 −40 4	5.81	0.037	2817	0.007
	L Temporal Mid (BA22)	−58 −28 4	4.20	0.570		
	L Supramarg (BA48)	−52 −38 28	4.29	0.517		
	L Temporal Sup/Rolandic Operc (BA41,48)	−42 −34 20	4.67	0.311		
E3	L Temporal Inf (BA20)	−46 4 −40	5.89	0.031	1073	0.033
	L Temporal pole (BA20)	−48 10 −32	4.65	0.322		
	L Front Inf/Orbitofr (IFG, BA45,47)	−48 25 −8	5.35	0.010		
E4	Cing Ant (vACC/VMPFC, BA11)	16 44 −6	5.67	0.050	1272	0.025
	Cing Ant (vACC, BA11)	−6 40 −2	4.48	0.408		
E5	Cing Mid (BA23)	−2 −16 38	5.68	0.050	1335	0.024
	Suppl motor (BA6)	4 −8 72	5.30	0.114		
	R Precentral (BA4)	20 −22 78	4.74	0.279		
E6	L Precentral (BA6)	−28 −26 72	5.13	0.150	1031	0.035
	L Postcenteral (BA3)	−22 −37 72	4.44	0.431		
	L Postcentral (BA4)	−26 −28 62	4.55	0.368		
E7	Cing Post (BA23)	−10 −48 32	4.46	0.420	415	0.121

*Explanation of symbols. MNI Coord, Montreal Neurological Institute coordinates, in mm; t, Student's t; p (peak), significance level; peak-level correction; k, cluster size, in 2 × 2 × 2 voxels; p (cl.): significance level, cluster-level correction. R, L, right, left; Ant, Post, Sup, Mid, Inf, anterior, posterior, superior, middle, inferior; Operc, operculum; Suppl, supplementary; Supramarg, supramarginal; Front, frontal; Orbitofr, orbitofrontal. IFG, inferior frontal gyrus; vACC, ventral anterior cingulate; VMPFC, ventromedial prefrontal cortex*.

## Discussion

One aim of the present study was to verify that the computerized version of the SST replicated known empirical findings of the original version. As in the original version, the computerized version of the SST was shown here to reveal a tendency to prefer positive sentences in the healthy, producing about 20 to 30% negative cognitions. Because participants are instructed to report a well-formed sentence, these data reflect a spontaneous tendency. Also the correlation with depressiveness scores as measured by common rating scales was replicated. This suggests that the computerized version may be used to empirically assess the tendency to form optimistic or pessimistic cognitions. Additional evidence that the SST provides empirical evidence for tendencies in the formation of cognitions is the finding that the self-relevant version of the sentences was significantly associated with a higher rate of positive sentences. The tendency to exceptionalist positive thinking when judgment concerns oneself has been described previously (Weinstein, 1980). A possible limitation of the technique we used in the development of the computerized version of the SST, however, is the reliance on the existence of sentences where the decisive word is located last, which may be language-specific.

The association of depressiveness with rates of positive sentence appeared to be selective to the theme of failure/success or pessimism/optimism, while the association in themes of loss and separation was not significant, notwithstanding the importance of loss and separation in the etiology of depression (Keller et al., 2007). It is not clear if this finding may be expected to be replicated in a more representative sample of individuals who have experienced loss. Because our sample was composed of young individuals, it is conceivable that our results may not be generalizable in this respect. We also found that other dimensions of affective psychopathology, such as anxiety and anhedonia, showed no specific association with rates of positive sentences. These dimensions, however, may not be relevant to subsyndromal depression, in contrast to the full depressive syndrome. Also in this case, the generalizability of our finding may be limited.

Additional evidence on the validity of the computerized version of the SST comes from the replication of a pattern of differential neural activation previously obtained with the original version of the test (Viviani et al., 2010). In that study, neutral sentences activated prefrontal areas such as DLPFC and MLPFC in comparison to emotional sentences. Emotional sentences, in turn, were characterized by a relative activation of vACC, the left supramarginal gyrus/Rolandic operculum, and (below the significance threshold) the inferior frontal gyrus. This pattern was replicated in the present study. Here, emotional sentences were also found to elicit a relative activation of the superior temporal gyrus, the anterior temporal pole, and of areas around the central gyrus. The more extensive activations in the present study may be attributed by a more precise evaluation of the effects of the scrambled sentence content at the time points when the sentences are presented (unlike the previous study).

A more general issue is what the SST may tell us about the nature of the tendency to prefer positive cognitions in the healthy. The distinctive feature of the SST on which this study focused is the emergence of tendencies in the formation of cognitions even in the absence of a cognitive load. Clearly, participants are fully aware of what sentence they choose, especially when no cognitive load is given, as it was the case here. However, the tendency to spontaneously prefer specific content emerges statistically across the sample. It seems difficult that a deliberate strategy in the selection of the sentences, such as one following the wish to adhere to social expectations, may be responsible for the pattern of results emerging from the data. The use of strategies cannot explain the individual differences associated with depressiveness scores observed across the sample nor the differential outcome of self-referential sentences. Participants have no knowledge of the typical rates of optimistic sentences in this test, or of what sentences other participants are likely to select. These findings are consistent with attainment of the original aims in the development of the SST, i.e., the empirical assessment of tendencies in the formation of spontaneous cognitions.

When the content of the sentence is emotional, as in the optimistic/pessimistic case investigated here, the SST potentially provides data to test models of emotion processing widely adopted in the clinical neurosciences. As in the original theoretical framework in which the SST was developed, a common tenet of these models is that an effortful cognitive process of executive nature influences choices made when participants are aware of their decisions. This control process antagonizes the tendency of emotional content to enter working memory in virtue of its emotional salience. Affective disorders or maladaptive forms of emotion regulation are modeled as a disturbance of the capacity of cognitive control processes to prevail (Phillips et al., 2003; Bishop et al., 2004; Ochsner and Gross, 2005; Siegle et al., 2007; Beck, 2008). There is now ample evidence that the neural substrates of cognitive control are active when explicitly regulating emotion processing (Ochsner et al., 2002; Compton, 2003; Phan et al., 2005; Banich et al., 2009), thus explaining changes in rates of negative cognitions observed under cognitive load. In the SST, positive sentences are preferentially chosen even when the negative targets are more salient than their positive counterparts (Viviani et al., 2010). Hence, some mechanism other than salience of targets must be responsible for their selection, and cognitive control is a candidate process for this mechanism.

However, the evidence does not unequivocally support this model. In the neuroimaging data, prefrontal areas associated with executive processes showed no increased recruitment when the content of the sentences was emotional. Increased recruitment of these areas would be expected if executive processes were recruited to exclude the more salient negative alternative, as in previous studies on explicit regulation. On the contrary, these areas were less active than when the content was neutral, replicating the findings of the previous study (Viviani et al., 2010). As that study showed, the relative lack of recruitment of these substrates was related to the spontaneous nature of avoidance of negative content. Neural substrates of executive function were activated if avoidance of negative content followed an explicit instruction of the experimenter. Furthermore, in that same study, there was no association between the rate of positive sentences in the spontaneous condition and individual differences in working memory capacity. Also the negative association between positive sentence formation rates and time of response found in the present study is not consistent with the intervention of cognitive control. When they have an influence, longer response times favor recruitment of executive processes (Finucane et al., 2000). Hence, if cognitive control is recruited to form positive cognitions, the formation rate of positive sentences should increase, not decrease, with longer response times.

In alternative to cognitive control, a possible explanation of these findings is that people spontaneously form positive cognitions because they prefer them. It is just more pleasant to think that the future is bright than dismal. The apparent triviality of this alternative explanation belies its potential theoretical relevance in explaining the influence of emotional states on cognition and the role of preference-formation in emotion regulation (Viviani, 2014; Berkman et al., 2016, 2017). Decisions made on the base of subjective preferences are based on sophisticated mechanisms that integrate past associative information on the appetitiveness and aversiveness of cues with current motivational states (value-based decision making, Rangel et al., 2008). The shift of brain activity toward ventral areas observed when the SST involves emotional sentences, and more specifically the involvement of vACC/VMPFC, is consistent with recruitment of areas evaluating the preference value of alternatives (Levy and Glimcher, 2012; Bartra et al., 2013; Rangel and Clithero, 2014) and the modulation of emotional processing (Etkin et al., 2006; Elliott et al., 2011). The relative activation of the SMG/Rolandic operculum in the emotional set may also be consistent with activation of neural substrates recruited by choice tasks involving emotional material (Adolphs et al., 1996). These findings are consistent with the hypothesis that the avoidance of negative material, even if more salient, may be under control of a mechanism coordinating motivational and associative processes characterizing spontaneous decisions, rather than executive processes (Viviani, 2014).

Ultimately, the SST exposes a more or less explicit but fundamental controversy on the relationship between behavioral decisions of which participants are aware and cognitive control/executive function. In a dual-process framework in which executive function biases access to working memory of content of varying intrinsic salience, deliberate choice follows from executive function alone. However, there are several situations in clinical experience when this explanatory model appears to be inapplicable (Messina et al., 2016). An alternative hypothesis is that executive function and motivational processes provide distinct but equally powerful sources of control of cognitions and behavior.

## Author contributions

RV: conceived study; designed research; contributed software for the experiment and data analysis; analyzed data; wrote manuscript. LD: designed research; acquired data; analyzed data. JB: acquired data; analyzed data. JS: designed research. PB: designed research. All: revised manuscript and approved final version.

### Conflict of interest statement

The authors declare that the research was conducted in the absence of any commercial or financial relationships that could be construed as a potential conflict of interest.

## Appendix

**Table A1:** Sentences used in the experiments.

**ID**	**Type**	**Self**	**Text**	**Pos**
A1i1	A	0	sind ihrer tot Freunde treu viele	0.85
A1i13	A	0	herzlich viele sich Eltern verhalten kühl	0.86
A1i2	A	0	glücklich oft Maria ist traurig ziemlich	0.56
A1i4	A	0	fühlt einsam sich Junge dieser wohl	0.59
A1i5	A	0	viele verloren Freunde gefunden er hat	0.81
A1i6	A	0	Zuversicht steckt Leben das voller Trauer	0.88
A1p1	A	1	tot sind viele Freunde meiner treu	0.81
A1p13	A	1	sich kühl verhalten Eltern meine herzlich	0.95
A1p2	A	1	oft bin glücklich ich traurig ziemlich	0.91
A1p4	A	1	oft wohl ich einsam mich fühle	0.53
A1p5	A	1	verloren ich Freunde habe viele gefunden	0.77
A1p6	A	1	Zuversicht voller Leben steckt Trauer mein	0.95
A2i10	A	0	erlebt Erfüllung man Beziehungen in Enttäuschung	0.76
A2i11	A	0	viele soziale Ablehnung Kinder Sicherheit erleben	0.42
A2i12	A	0	verloren Anna Liebe hat gefunden ihre	0.84
A2i7	A	0	es Streit zwischen Einigkeit ihnen gibt	0.69
A2i8	A	0	Erlebnisse voller Vergangenheit die war Einsamkeit	0.69
A2i9	A	0	wiedersehen ihren Anna Freund wird vermissen	0.49
A2p10	A	1	Enttäuschung in erlebe Beziehungen ich Erfüllung	0.80
A2p11	A	1	soziale viele Sicherheit erlebe Ablehnung ich	0.83
A2p12	A	1	verloren ich Liebe habe gefunden meine	0.77
A2p7	A	1	Streit gibt uns zwischen es Einigkeit	0.72
A2p8	A	1	meine Erlebnisse war Vergangenheit voller Einsamkeit	0.70
A2p9	A	1	ich wiedersehen Freund vermissen werde meinen	0.58
O1i1	O	0	irgendwann dieser reich Mann arbeitslos wird	0.56
O1i3	O	0	Sorgen eher Zukunft die Freude macht	0.60
O1i4	O	0	Erfolg hat man gewöhnlich für Misserfolg	0.85
O1i5	O	0	meisten verpasst Chancen genutzt die werden	0.63
O1i6	O	0	enttäuscht werden Wünsche wichtige öfters erfüllt	0.79
O1p1	O	1	reich werde irgendwann ich auch arbeitslos	0.91
O1p3	O	1	die Sorgen macht Zukunft mir Freude	0.53
O1p4	O	1	Erfolg habe gewöhnlich ich für Misserfolg	0.91
O1p5	O	1	viele verpasst Chancen genutzt ich habe	0.67
O1p6	O	1	erfüllt öfters Wünsche meine enttäuscht werden	0.79
O2i10	O	0	sinnlos heutzutage ist Studium ein sinnvoll	0.81
O2i11	O	0	bestimmt prima Bewerbung enttäuschend diese verläuft	0.79
O2i13	O	0	macht müde meist Arbeit die glücklich	0.56
O2i7	O	0	erreichen man Ziele wichtige kann verfehlen	0.91
O2i8	O	0	ist es versagen leicht bestehen zu	0.75
O2i9	O	0	rosig eher Zukunft die düster wird	0.84
O2p10	O	1	ist sinnvoll eher Studium mein sinnlos	0.91
O2p11	O	1	meine prima Bewerbung enttäuschend bestimmt verläuft	0.79
O2p13	O	1	macht meine glücklich Arbeit müde mich	0.67
O2p7	O	1	verfehlen werde meine Ziele ich erreichen	0.98
O2p8	O	1	versagen werde mal ich wieder bestehen	0.84
O2p9	O	1	düster wird Zukunft eher rosig meine	0.88
N1i1	N	0	gehört hat das er oft gesehen	
N1i2	N	0	Kaffee beim trinkt Frühstück Tee Katharina	
N1i4	N	0	im die Schnee spielen Sand Kinder	
N1i6	N	0	Motorrad mit Michael fährt dem Fahrrad	
N1p1	N	1	das gehört habe ich öfters gesehen	
N1p2	N	1	beim ich Tee trinke Kaffee Frühstück	
N1p4	N	1	Schnee spiele ich im immer Sand	
N1p6	N	1	Motorrad dem ich mit fahre Fahrrad	
N2i10	N	0	Frühling ist ihr Geburtstag im Sommer	
N2i11	N	0	neue Grundschule Johanna besucht die Hochschule	
N2i12	N	0	dem vor Geschäft steht Postamt Michael	
N2i7	N	0	Fußball über weiß er viel Handball	
N2i8	N	0	ist hellblau Strumpfhose dunkelblau diese neue	
N2i9	N	0	gemütlich ist Sofa sehr bequem dieses	
N2p10	N	1	Frühling mein im Geburtstag ist Sommer	
N2p11	N	1	Hochschule neue besuche ich Grundschule die	
N2p12	N	1	dem Postamt stehe Geschäft ich gegenüber	
N2p7	N	1	Fußball mich ich für interessiere Handball	
N2p8	N	1	ist neue dunkelblau Strumpfhose hellblau meine	
N2p9	N	1	mein gemütlich ist Sofa sehr bequem	
T1	S	0	enthält Dokument eine dieses Überschrift Unterschrift	0.85
Ti2	S	0	Kino sie Theater zusammen ins gingen	0.94
Tp2	S	1	Kino wir Theater zusammen ins gingen	0.94
Tp3	S	1	suche ich neues Auto ein Haus	0.85
Ti4	S	0	dünn dick das sehr ist Buch	0.92
Tp4	S	1	dünn dick mein sehr ist Buch	0.92
Tp5	S	1	Zug Bus ich ersten nehme den	0.90
Ti5	S	0	Zug Bus Hannelore ersten nimmt den	0.90

*Explanation of symbols: A, attachment/loss scrambled sentences; O, optimism/pessimism scrambled sentences; N, neutral scrambled sentences; S, sentinel scrambled sentences; Self, self-referential or non self-referential; Pos, frequency of formation of a positive sentence/correct sentence (for sentinel trials)*.
